# Supplementary material for: Differential efficacies of Cas nucleases on microsatellites involved in human disorders and associated off-target mutations
Source: Nucleic Acids Res. 2021 Jul 7;49(14):8120–34. doi: 10.1093/nar/gkab569 (PMC8373144; doi:10.1093/nar/gkab569)
Supplement: gkab569_Supplemental_Files [file gkab569_supplemental_files.zip › Supplementary legends_final.docx]

**Supplemental Figures**

**Supplemental Figure S1: DSB-repair efficacy in mutant backgrounds. A:** *rad51*Δ strain. Top: dot plot graphs of GFP-positive cells at different time points. Green cells are boxed. Middle: Percentage of GFP-positive cells in glucose and galactose media at each time point. The non-repeated sequence (NR) is shown as a comparison. Bottom: Southern blot analysis of DSB efficacy at several time points. Recombinant molecules and DSB signals are quantified and shown below as a percentage of the total signal in the lane. **B:** *dnl4*Δ strain. Same as above. **C:** *pol32*Δ strain. Same as above, expect that the time course was performed on a longer time period (see Materials & Methods). Note that the DSB signal is undetectable at the time points analyzed.

**Supplemental Figure S2: Histogram of GFP-positive cell percentages for each repeat and each nuclease.** Galactose condition is highlighted in green and glucose condition in black for each of the four time points. Each experiment was performed 3-8 times, depending on the strain. Error bars are standard errors.

**Supplemental Figure S3: Flow cytometry at 36 hours for each repeat.** For each repeat (horizontal) the corresponding dot plot is shown for each nuclease (vertical). Gates are drawn to separate recombined from non-recombined populations. Note that more cells were analyzed for SaCas9 and experiments were run on a different cytometer.

**Supplemental Figure S4: Southern blots and quantifications.** For each repeat, a Southern blot of an induction time course over 12 hours is shown above quantification graphs. Recombination and DSB percentages were calculated as fractions of the total signal in each lane. Note that no reliable quantification could be performed for *Sp*Cas9-D10A on the TGGAA repeat, due to the presence of two very close bands corresponding in size to the DSB band.

**Supplemental Figure S5:** **sgRNA and protein levels. A: sgRNA** expression levels measured by Northern blot. Top: Northern blot was hybridized with a SpCas9 sgRNA scaffold probe. The same Northern blots were rehybridized with a control *SNR44* probe, corresponding to a snoRNA gene. Bottom: Signals of SpCas9 sgRNA and *SNR44* were quantified and their ratios compared to nuclease efficacy measured by the percentage of GFP-positive cells at 36 hours. **B:** Nuclease expression levels measured by Western blot. Top: blots were successively hybridized with Cas-specific antibodies and Zwf1p antibody. Ratios of Cas/Zwf1 signals are shown below the blots. Bottom: GFP+ cell percentage (NR at 36 hrs) as a function of sgRNA levels.

**Supplemental Figure S6: Resection analysis of SpCas9 and FnCpf1 induced double-strand breaks.** *Sty*I restriction sites located on each side of the DSB site are indicated. Only yeast strains for which a DSB was detectable at 12 hour post-nuclease induction were analyzed. Resection at NR was set at 100% to normalize the data. Lower resection values indicate resection inhibition.

**Supplemental Figure S7: Evidence for *in vivo* DSBs generated by nickases.** Top: Southern blot of DNA prepared in agarose plugs for three strains (Non-repeated, CGG and GAA repeats) after 4, 8 and 12 hours of nuclease induction. Parental, recombinant and DSB bands are indicated. DSBs are visible for CGG and GAA strains, increasing with time. The asterisk indicates a migration artefact commonly observed when overloading DNA in a lane. Bottom: The same experiment was performed but DNA was prepared by the standard protocol. The DSB was faintly detected at similar time points. Note that for the control non-repeated strain, some DSB signal is detected at the T0 time point only, suggesting that it could correspond to mechanically broken DNA molecules.

**Supplemental Figure S8: dsODN genome coverage.** Each horizontal line represents a whole genome, in which each chromosome is separated by a dashed line and identified by a roman number. Red arrows point to regions enriched for the dsODN tag (rDNA and mitochondrial DNA).

**Supplemental Figure S9:** Mutations identified in each of the three off-target sequences. Deletions are indicated by a red Δ, insertions are in blue, and base substitutions in green. Triplets different from the microsatellite consensus are grey.
