# Supplementary material for: Differential efficacies of Cas nucleases on microsatellites involved in human disorders and associated off-target mutations
Source: Nucleic Acids Res. 2021 Jul 7;49(14):8120–34. doi: 10.1093/nar/gkab569 (PMC8373144; doi:10.1093/nar/gkab569)
Supplement: gkab569_Supplemental_Files [file gkab569_supplemental_files.zip › Supplementary Figures1-9.zip!/Supplemental Figure S1.pdf]

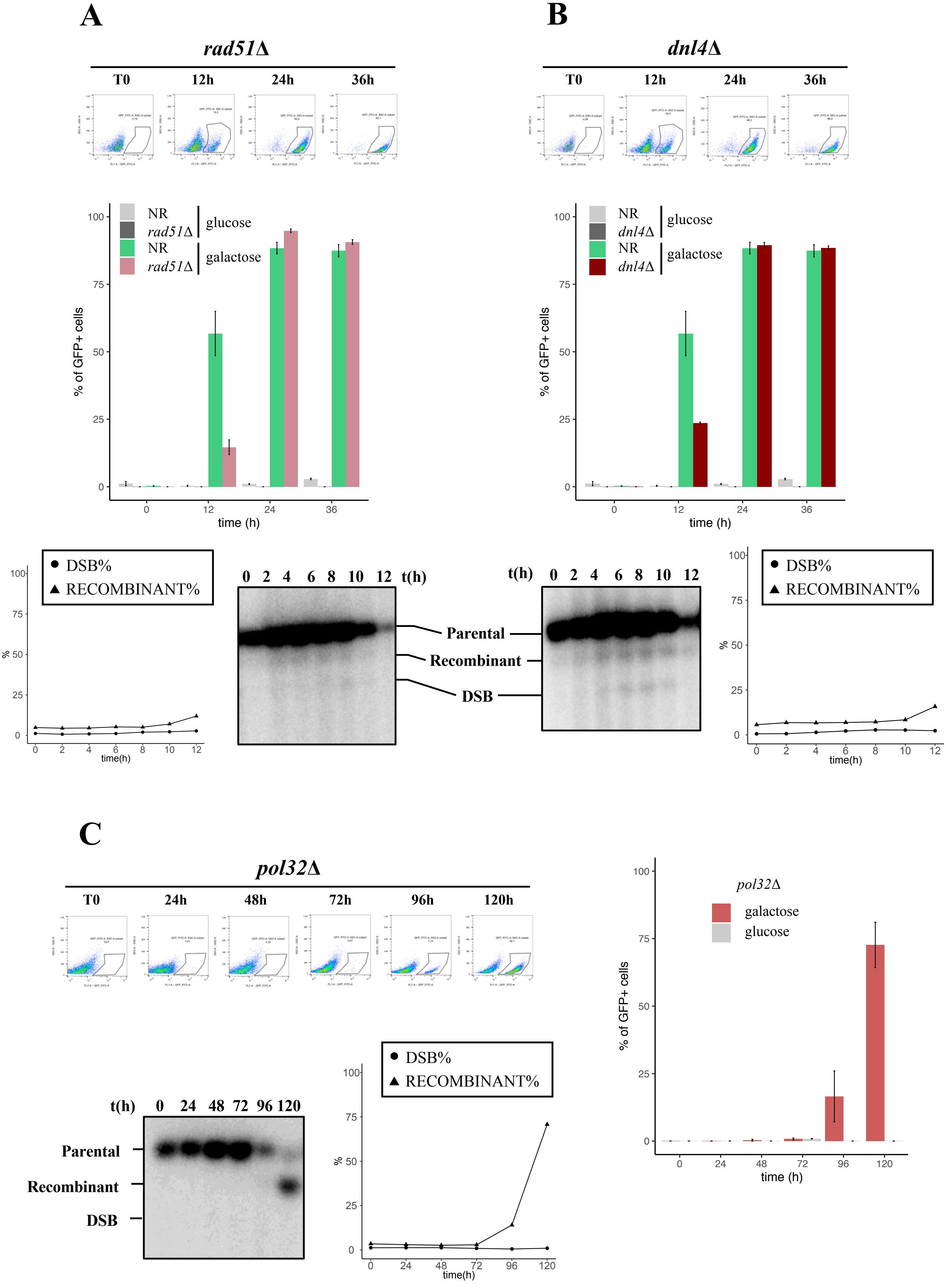
A
rad51Δ
T0
12h
24h
36h
NR
rad51Δ
glucose
NR
rad51Δ
galactose
% of GFP+ cells
100
75
50
25
0
0
12
24
36
time (h)
B
dnl4Δ
T0
12h
24h
36h
NR
dnl4Δ
glucose
NR
dnl4Δ
galactose
% of GFP+ cells
time (h)
DSB%
RECOMBINANT%
%
time(h)
0 2 4 6 8 10 12 t(h)
t(h) 0 2 4 6 8 10 12
Parental
Recombinant
DSB
C
pol32Δ
T0
24h
48h
72h
96h
120h
t(h) 0 24 48 72 96 120
Parental
Recombinant
DSB
DSB%
RECOMBINANT%
%
0
24
48
72
96
120
time(h)
pol32Δ
galactose
glucose
% of GFP+ cells
time (h)
