# Supplementary material for: Differential efficacies of Cas nucleases on microsatellites involved in human disorders and associated off-target mutations
Source: Nucleic Acids Res. 2021 Jul 7;49(14):8120–34. doi: 10.1093/nar/gkab569 (PMC8373144; doi:10.1093/nar/gkab569)
Supplement: gkab569_Supplemental_Files [file gkab569_supplemental_files.zip › Supplementary Figures1-9.zip!/Supplemental Figure S2_revised.pdf]

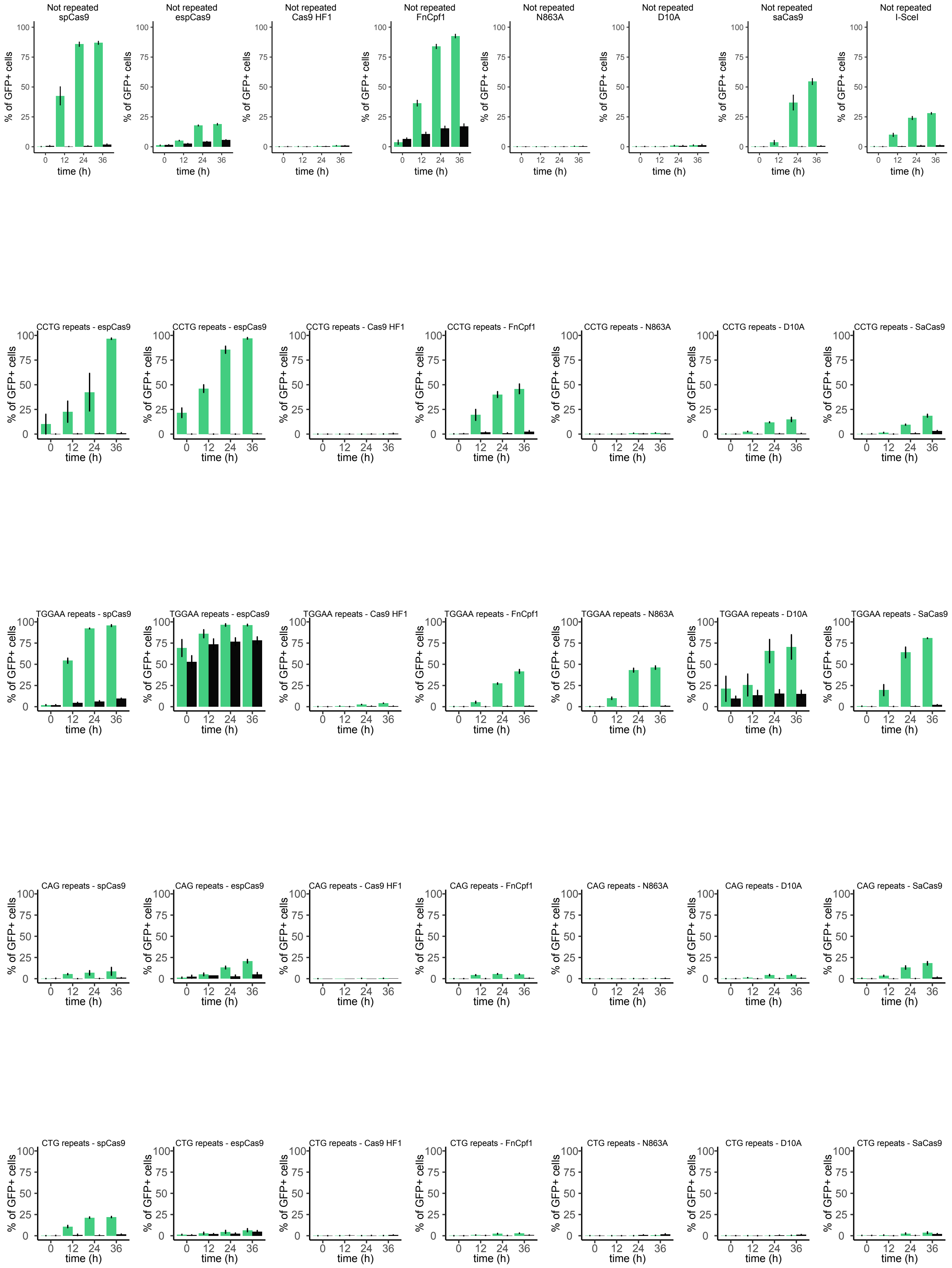
Not repeated spCas9
Not repeated espCas9
Not repeated Cas9 HF1
Not repeated FnCpf1
Not repeated N863A
Not repeated D10A
Not repeated saCas9
Not repeated I-SceI
CCTG repeats - espCas9
CCTG repeats - espCas9
CCTG repeats - Cas9 HF1
CCTG repeats - FnCpf1
CCTG repeats - N863A
CCTG repeats - D10A
CCTG repeats - SaCas9
TGGAA repeats - spCas9
TGGAA repeats - espCas9
TGGAA repeats - Cas9 HF1
TGGAA repeats - FnCpf1
TGGAA repeats - N863A
TGGAA repeats - D10A
TGGAA repeats - SaCas9
CAG repeats - spCas9
CAG repeats - espCas9
CAG repeats - Cas9 HF1
CAG repeats - FnCpf1
CAG repeats - N863A
CAG repeats - D10A
CAG repeats - SaCas9
CTG repeats - spCas9
CTG repeats - espCas9
CTG repeats - Cas9 HF1
CTG repeats - FnCpf1
CTG repeats - N863A
CTG repeats - D10A
CTG repeats - SaCas9
% of GFP+ cells
0
25
50
75
100
time (h)
0
12
24
36

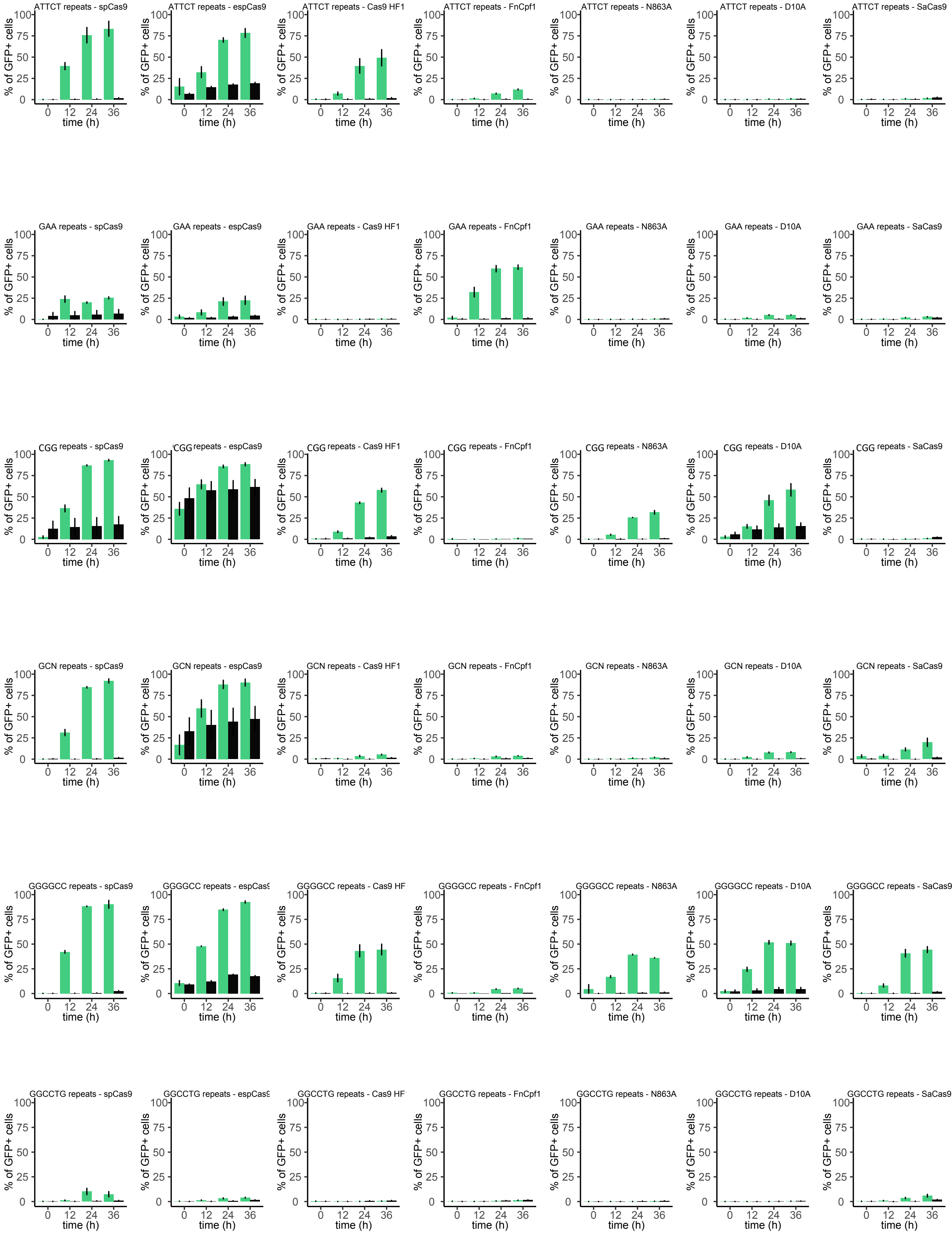
ATTCT repeats - spCas9
ATTCT repeats - espCas9
ATTCT repeats - Cas9 HF1
ATTCT repeats - FnCpf1
ATTCT repeats - N863A
ATTCT repeats - D10A
ATTCT repeats - SaCas9
GAA repeats - spCas9
GAA repeats - espCas9
GAA repeats - Cas9 HF1
GAA repeats - FnCpf1
GAA repeats - N863A
GAA repeats - D10A
GAA repeats - SaCas9
CGG repeats - spCas9
CGG repeats - espCas9
CGG repeats - Cas9 HF1
CGG repeats - FnCpf1
CGG repeats - N863A
CGG repeats - D10A
CGG repeats - SaCas9
GCN repeats - spCas9
GCN repeats - espCas9
GCN repeats - Cas9 HF1
GCN repeats - FnCpf1
GCN repeats - N863A
GCN repeats - D10A
GCN repeats - SaCas9
GGGGCC repeats - spCas9
GGGGCC repeats - espCas
GGGGCC repeats - Cas9 HF
GGGGCC repeats - FnCpf1
GGGGCC repeats - N863A
GGGGCC repeats - D10A
GGGGCC repeats - SaCas9
GGCCTG repeats - spCas9
GGCCTG repeats - espCas
GGCCTG repeats - Cas9 HF
GGCCTG repeats - FnCpf1
GGCCTG repeats - N863A
GGCCTG repeats - D10A
GGCCTG repeats - SaCas9
% of GFP+ cells
0
25
50
75
100
time (h)
0
12
24
36
