# Supplementary material for: Differential efficacies of Cas nucleases on microsatellites involved in human disorders and associated off-target mutations
Source: Nucleic Acids Res. 2021 Jul 7;49(14):8120–34. doi: 10.1093/nar/gkab569 (PMC8373144; doi:10.1093/nar/gkab569)

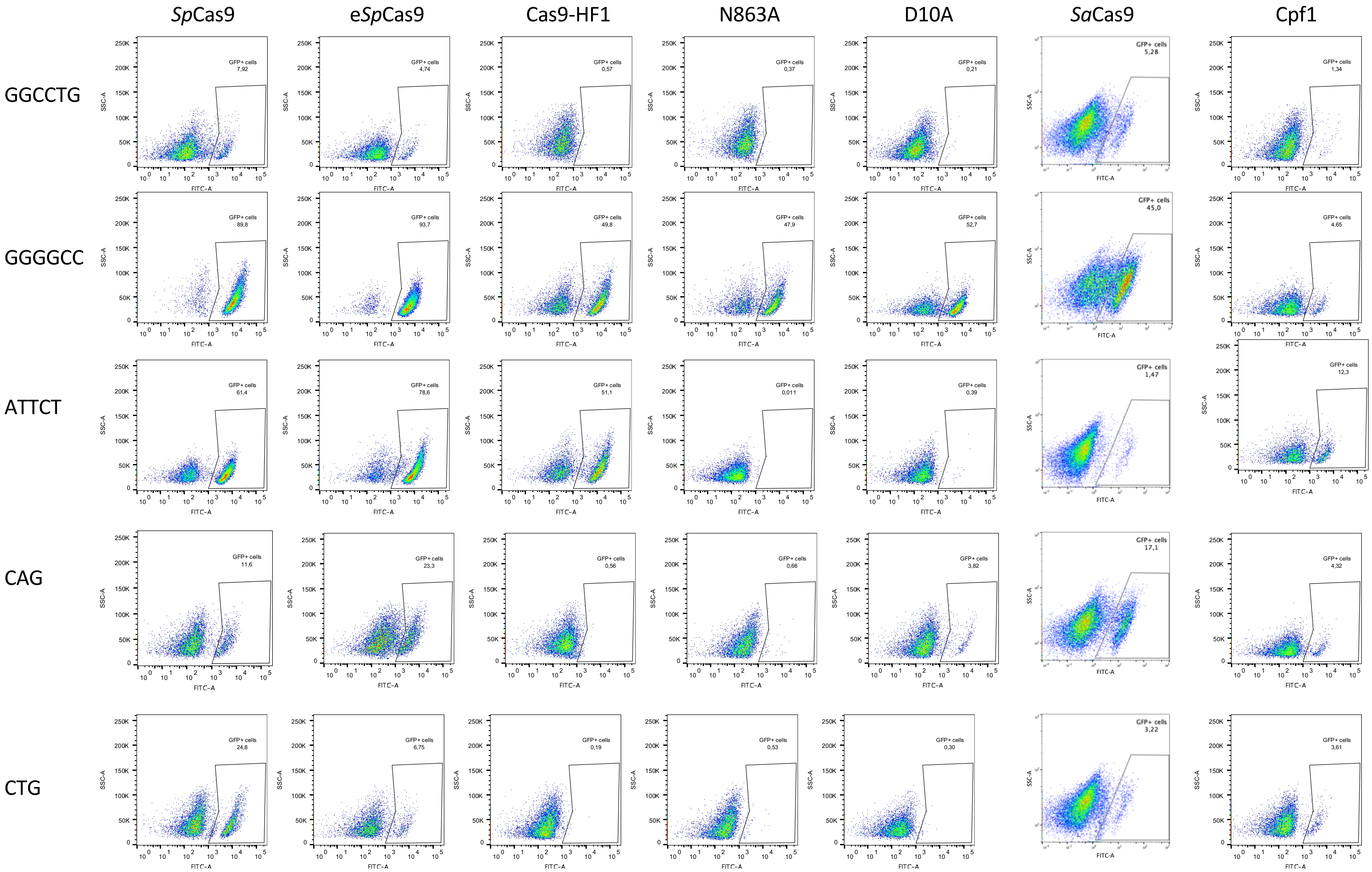

SpCas9
eSpCas9
Cas9-HF1
N863A
D10A
SaCas9
Cpf1
GGCCTG
GFP+ cells 7,92
GFP+ cells 4,74
GFP+ cells 0,57
GFP+ cells 0,37
GFP+ cells 0,21
GFP+ cells 5,28
GFP+ cells 1,34
GGGGCC
GFP+ cells 89,8
GFP+ cells 93,7
GFP+ cells 49,8
GFP+ cells 47,9
GFP+ cells 52,7
GFP+ cells 45,0
GFP+ cells 4,65
ATTCT
GFP+ cells 61,4
GFP+ cells 78,6
GFP+ cells 51,1
GFP+ cells 0,011
GFP+ cells 0,39
GFP+ cells 1,47
GFP+ cells 12,3
CAG
GFP+ cells 11,6
GFP+ cells 23,3
GFP+ cells 0,56
GFP+ cells 0,66
GFP+ cells 3,82
GFP+ cells 17,1
GFP+ cells 4,32
CTG
GFP+ cells 24,8
GFP+ cells 6,75
GFP+ cells 0,19
GFP+ cells 0,53
GFP+ cells 0,30
GFP+ cells 3,22
GFP+ cells 3,61
SSC-A
FITC-A

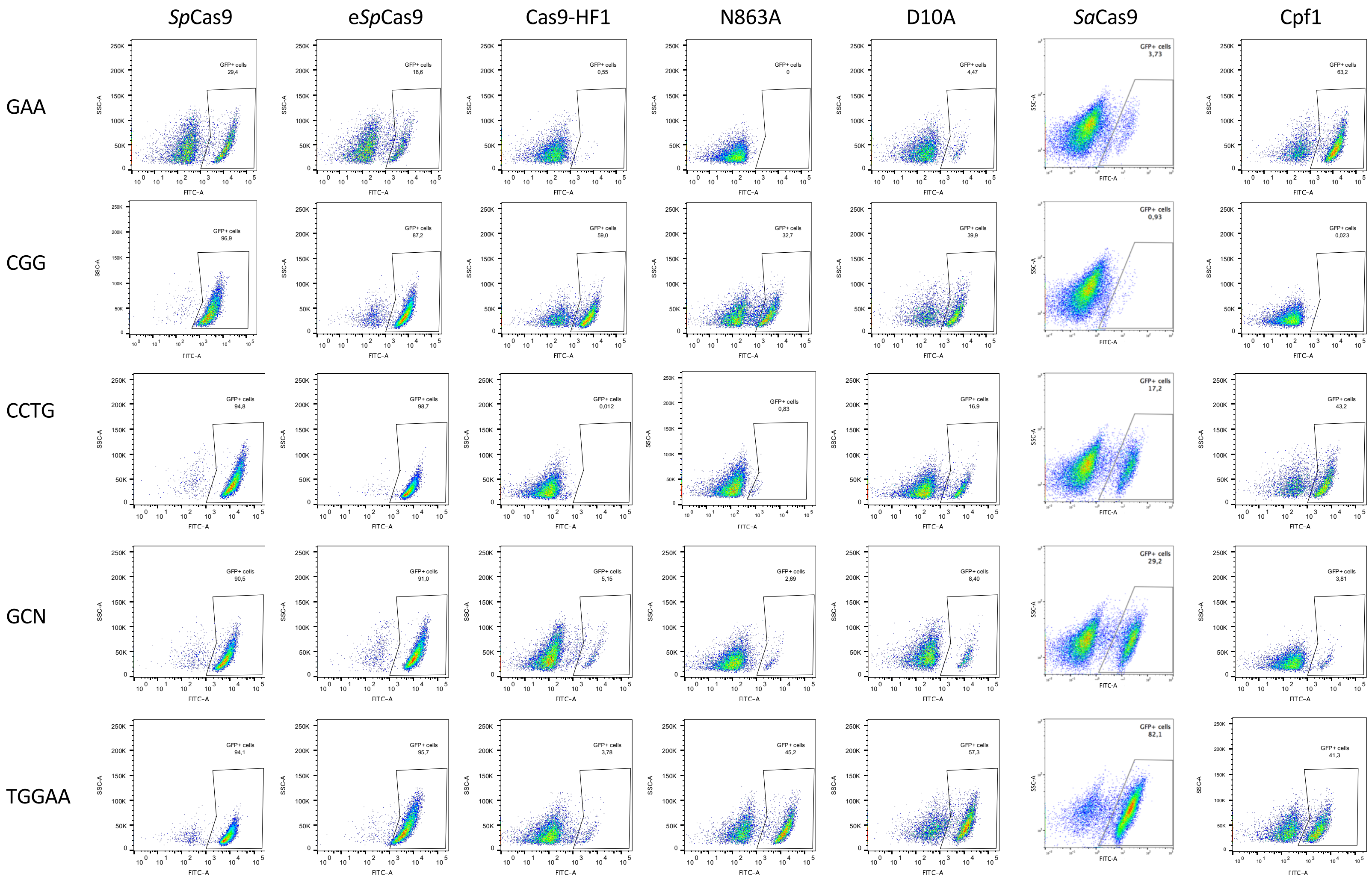
SpCas9
eSpCas9
Cas9-HF1
N863A
D10A
SaCas9
Cpf1
GAA
CGG
CCTG
GCN
TGGAA
GFP+ cells
29,4
18,6
0,55
0
4,47
3,73
63,2
96,9
87,2
59,0
32,7
39,9
0,93
0,023
94,8
98,7
0,012
0,83
16,9
17,2
43,2
90,5
91,0
5,15
2,69
8,40
29,2
3,81
94,1
95,7
3,78
45,2
57,3
82,1
41,3
SSC-A
FITC-A

Not repeated (NR)

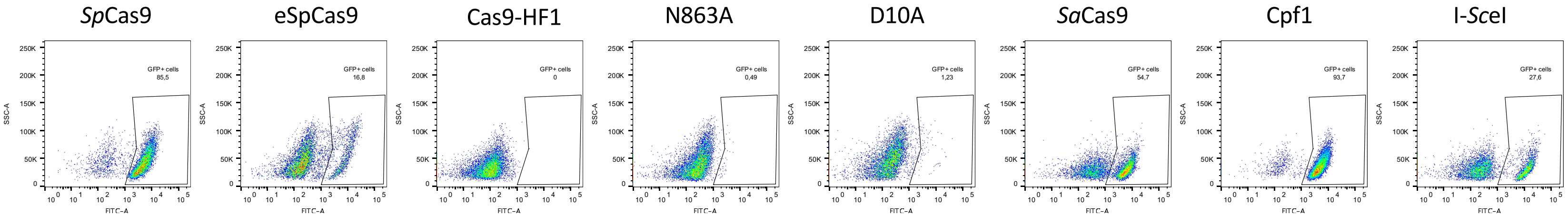

Supplement: gkab569_Supplemental_Files [file gkab569_supplemental_files.zip › Supplementary Figures1-9.zip!/Supplemental Figure S3_revised.pdf]
