# Supplementary material for: Differential efficacies of Cas nucleases on microsatellites involved in human disorders and associated off-target mutations
Source: Nucleic Acids Res. 2021 Jul 7;49(14):8120–34. doi: 10.1093/nar/gkab569 (PMC8373144; doi:10.1093/nar/gkab569)
Supplement: gkab569_Supplemental_Files [file gkab569_supplemental_files.zip › Supplementary Figures1-9.zip!/Supplemental Figure S4_revised.pdf]

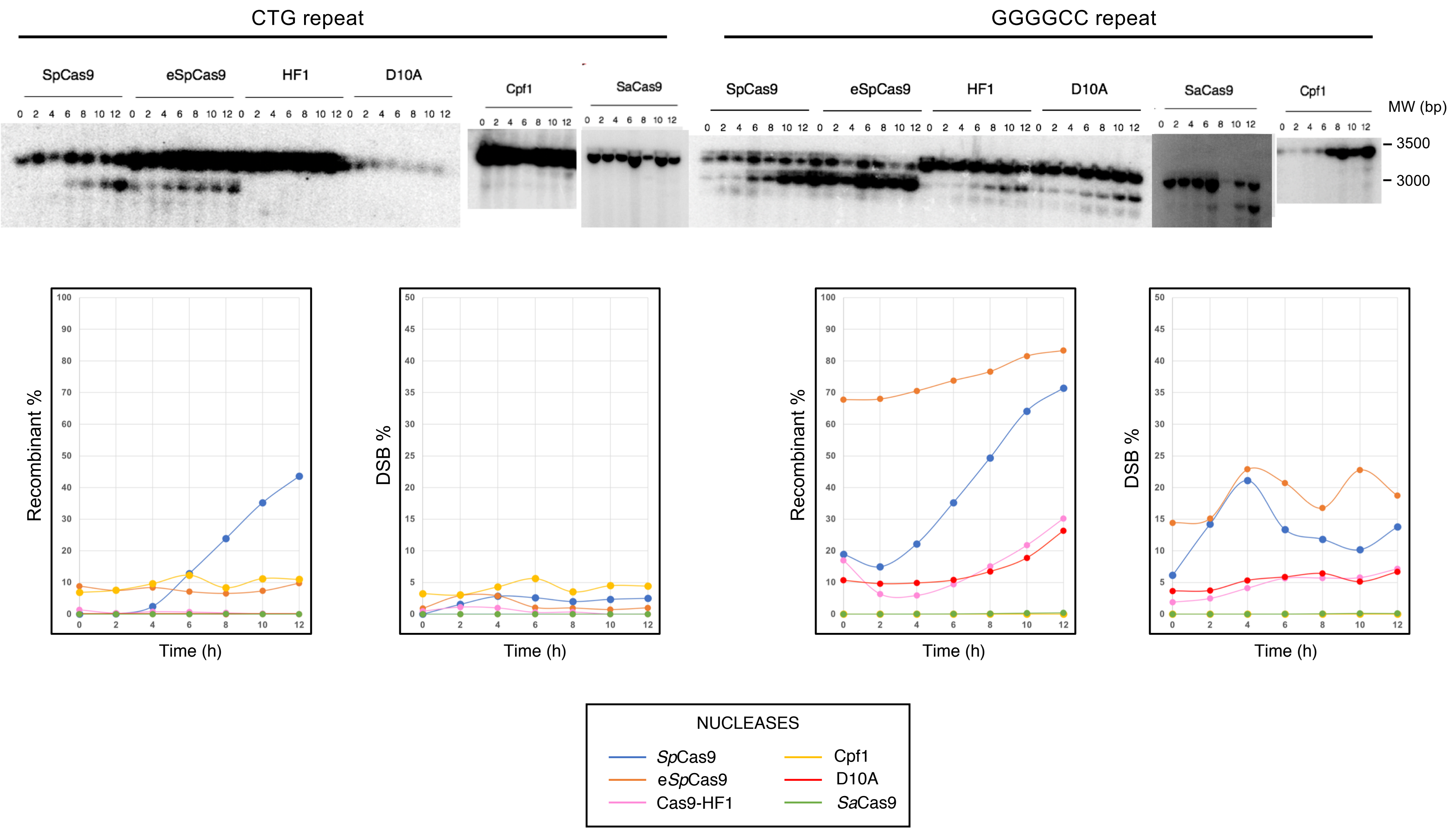

CTG repeat
GGGGCC repeat
SpCas9
eSpCas9
HF1
D10A
Cpf1
SaCas9
0 2 4 6 8 10 12
MW (bp)
3500
3000
Recombinant %
DSB %
Time (h)
NUCLEASES
SpCas9
eSpCas9
Cas9-HF1
Cpf1
D10A
SaCas9

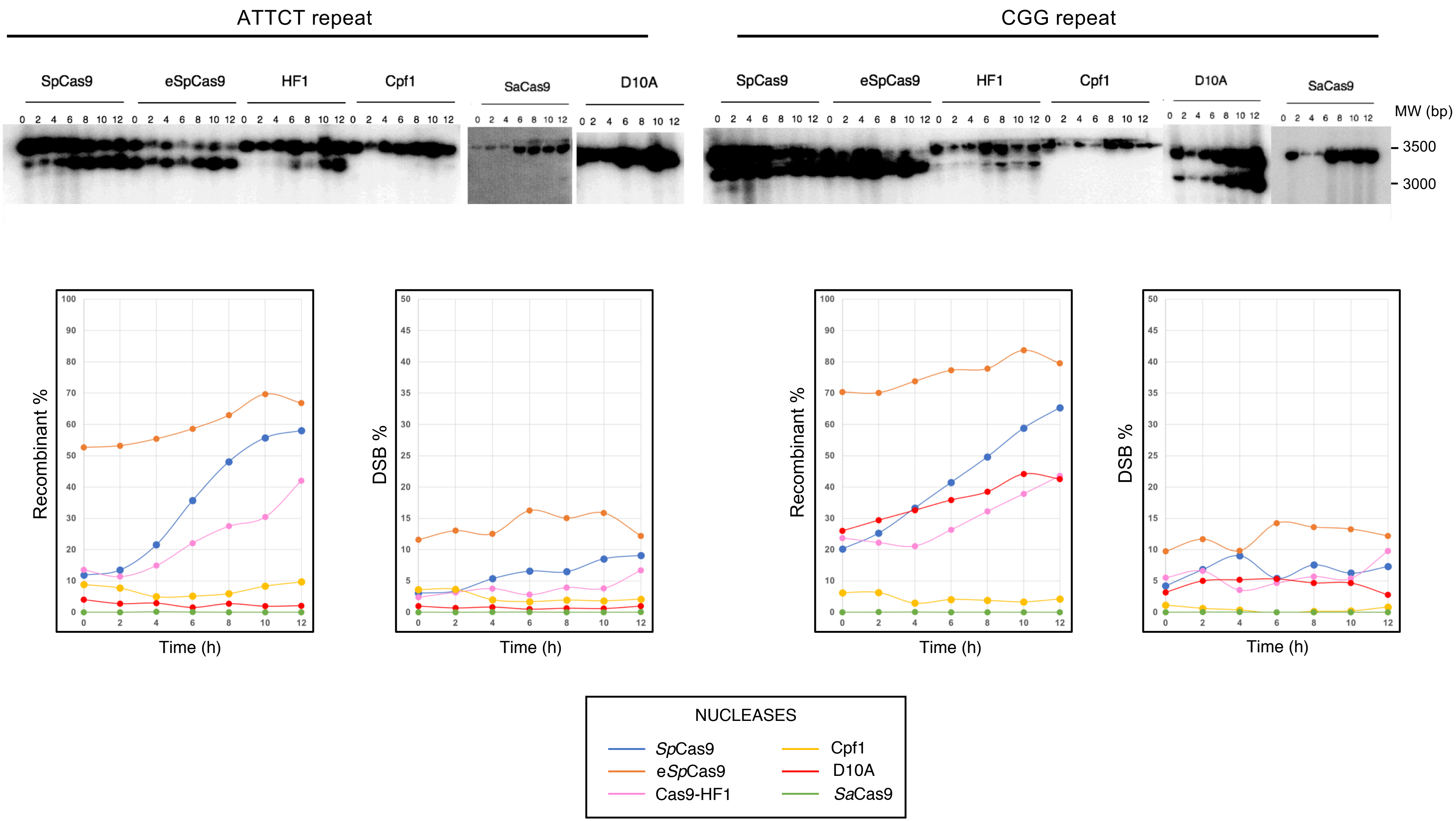

ATTCT repeat
CGG repeat
SpCas9
eSpCas9
HF1
Cpf1
SaCas9
D10A
0 2 4 6 8 10 12
MW (bp)
3500
3000
Recombinant %
DSB %
Time (h)
NUCLEASES
SpCas9
eSpCas9
Cas9-HF1
Cpf1
D10A
SaCas9

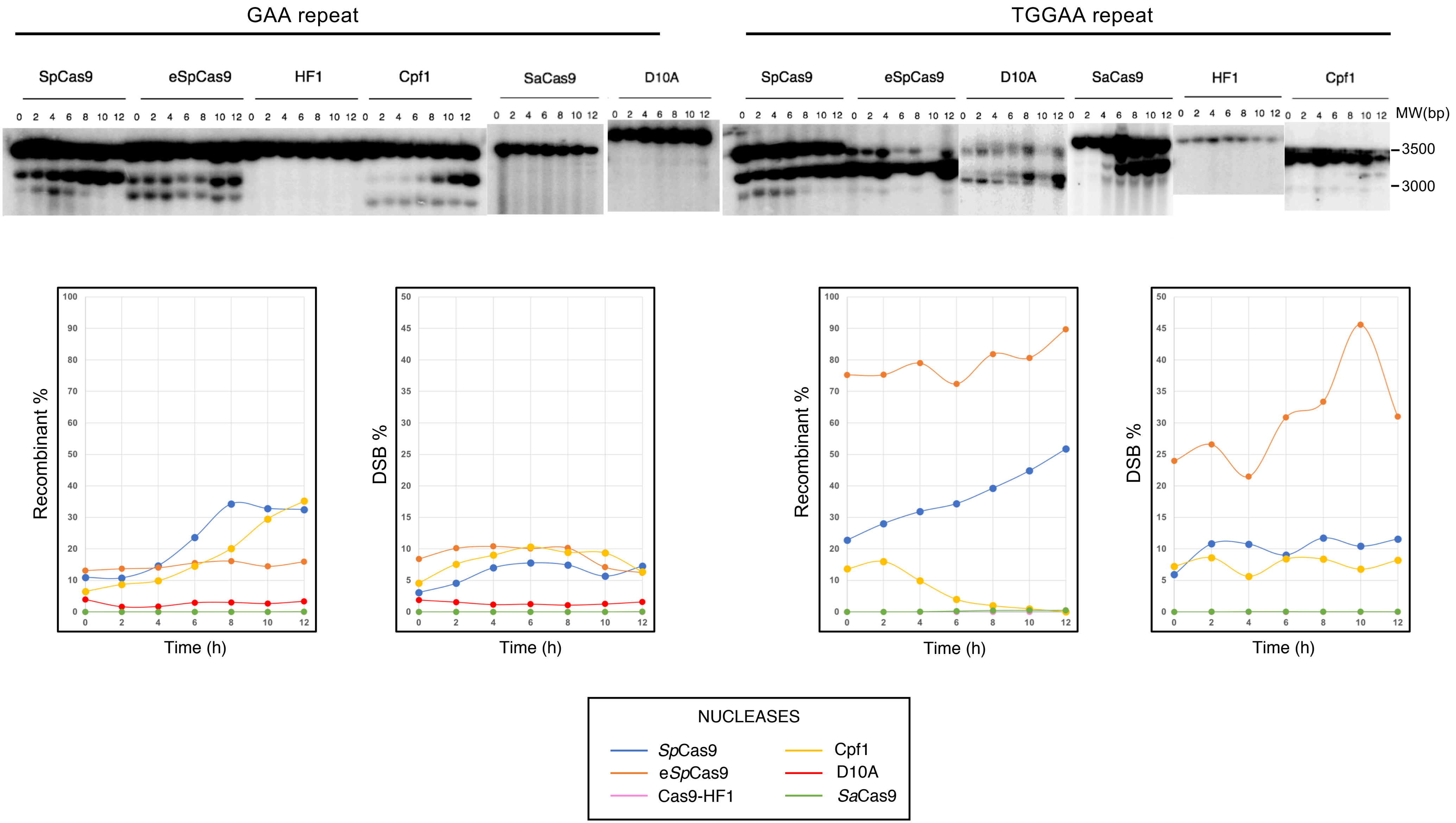
GAA repeat
TGGAA repeat
SpCas9
eSpCas9
HF1
Cpf1
SaCas9
D10A
MW(bp)
3500
3000
Recombinant %
DSB %
Time (h)
NUCLEASES
SpCas9
eSpCas9
Cas9-HF1
Cpf1
D10A
SaCas9

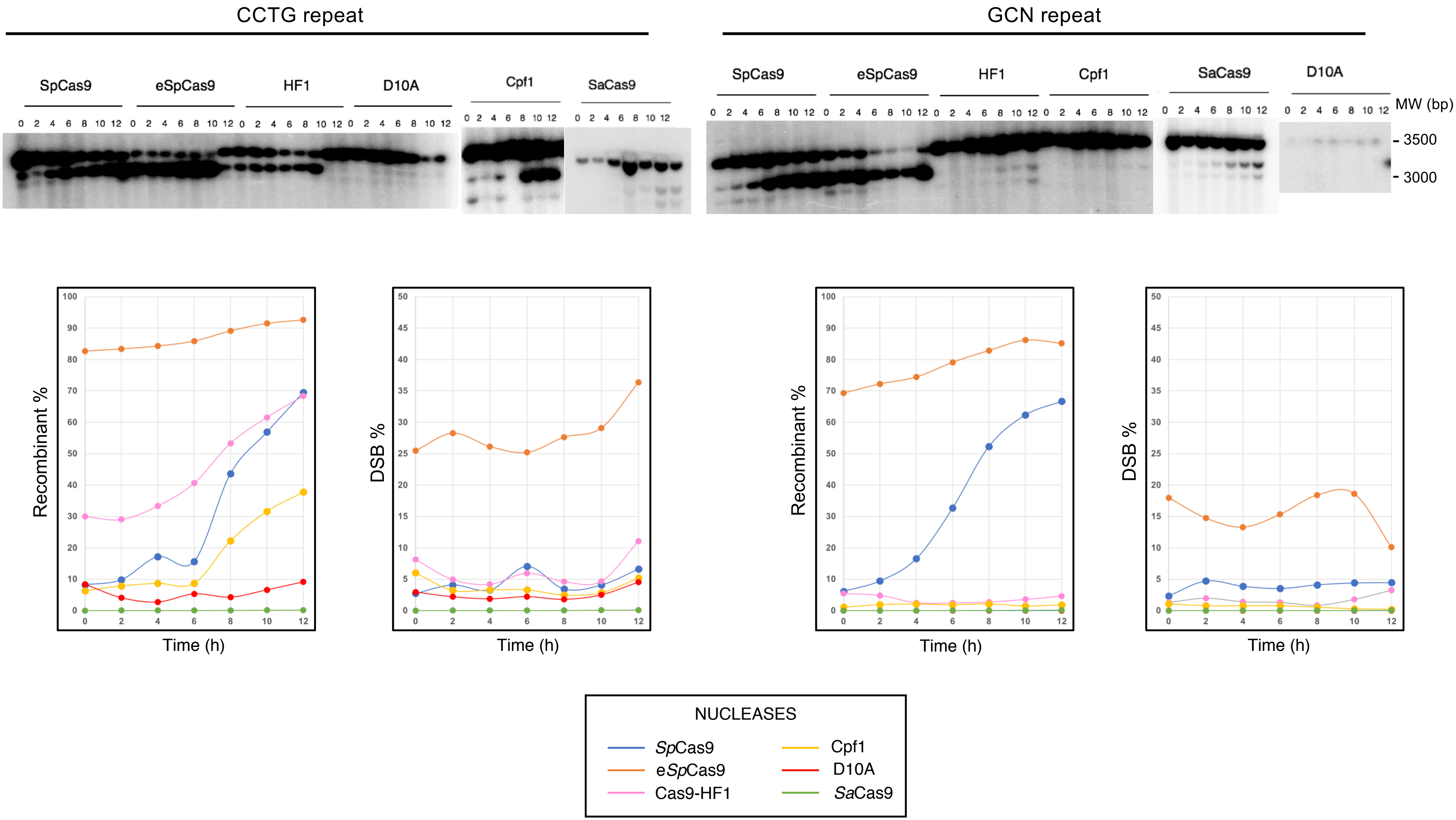
CCTG repeat
GCN repeat
SpCas9
eSpCas9
HF1
D10A
Cpf1
SaCas9
0 2 4 6 8 10 12
MW (bp)
3500
3000
Recombinant %
DSB %
Time (h)
NUCLEASES
SpCas9
eSpCas9
Cas9-HF1
Cpf1
D10A
SaCas9

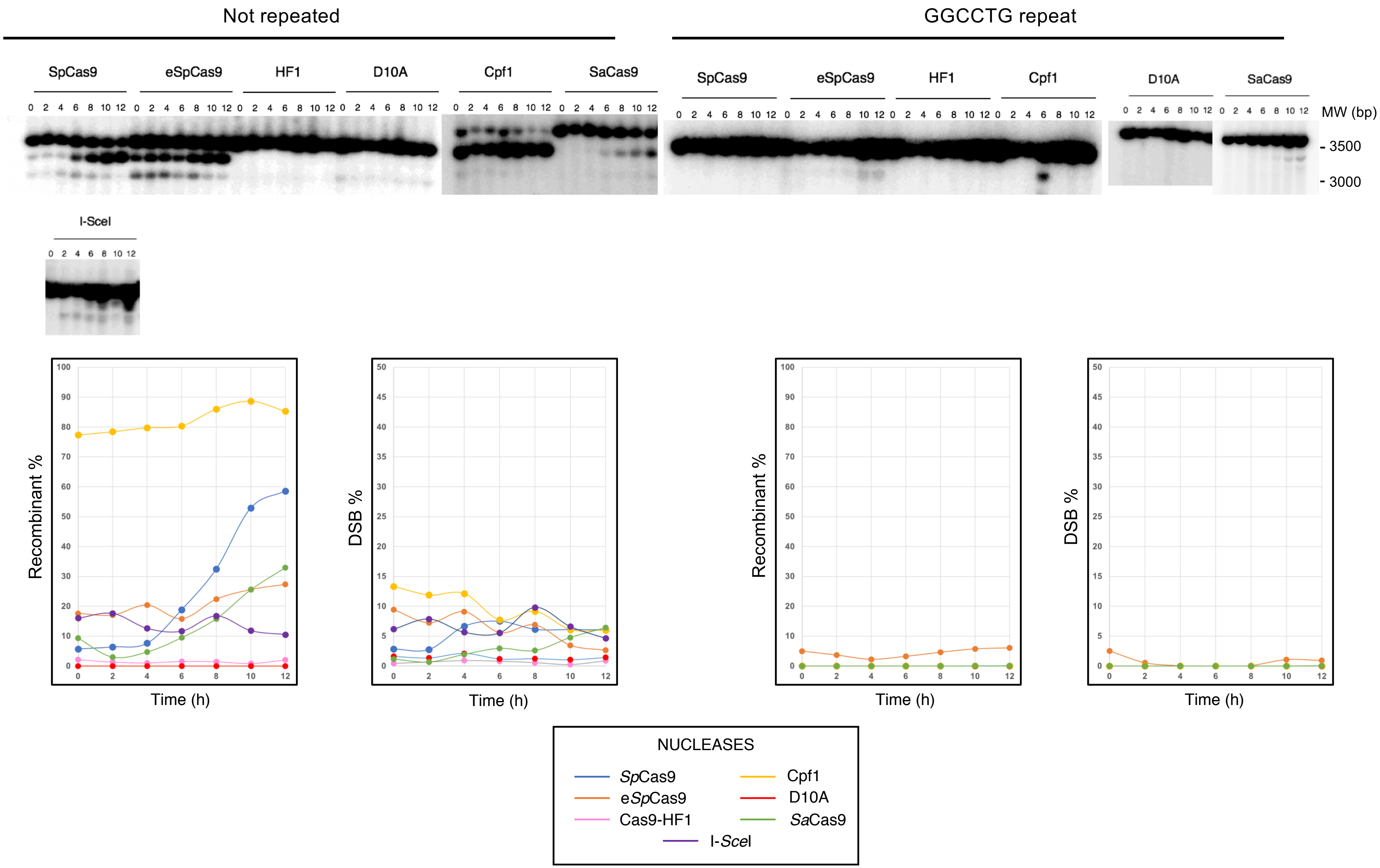

Not repeated
GGCCTG repeat
SpCas9
eSpCas9
HF1
D10A
Cpf1
SaCas9
SpCas9
eSpCas9
HF1
Cpf1
D10A
SaCas9
MW (bp)
3500
3000
I-SceI
0 2 4 6 8 10 12
Recombinant %
DSB %
Time (h)
NUCLEASES
SpCas9
eSpCas9
Cas9-HF1
I-SceI
Cpf1
D10A
SaCas9
