# Supplementary material for: Differential efficacies of Cas nucleases on microsatellites involved in human disorders and associated off-target mutations
Source: Nucleic Acids Res. 2021 Jul 7;49(14):8120–34. doi: 10.1093/nar/gkab569 (PMC8373144; doi:10.1093/nar/gkab569)
Supplement: gkab569_Supplemental_Files [file gkab569_supplemental_files.zip › Supplementary Figures1-9.zip!/Supplemental Figure S5.pdf]

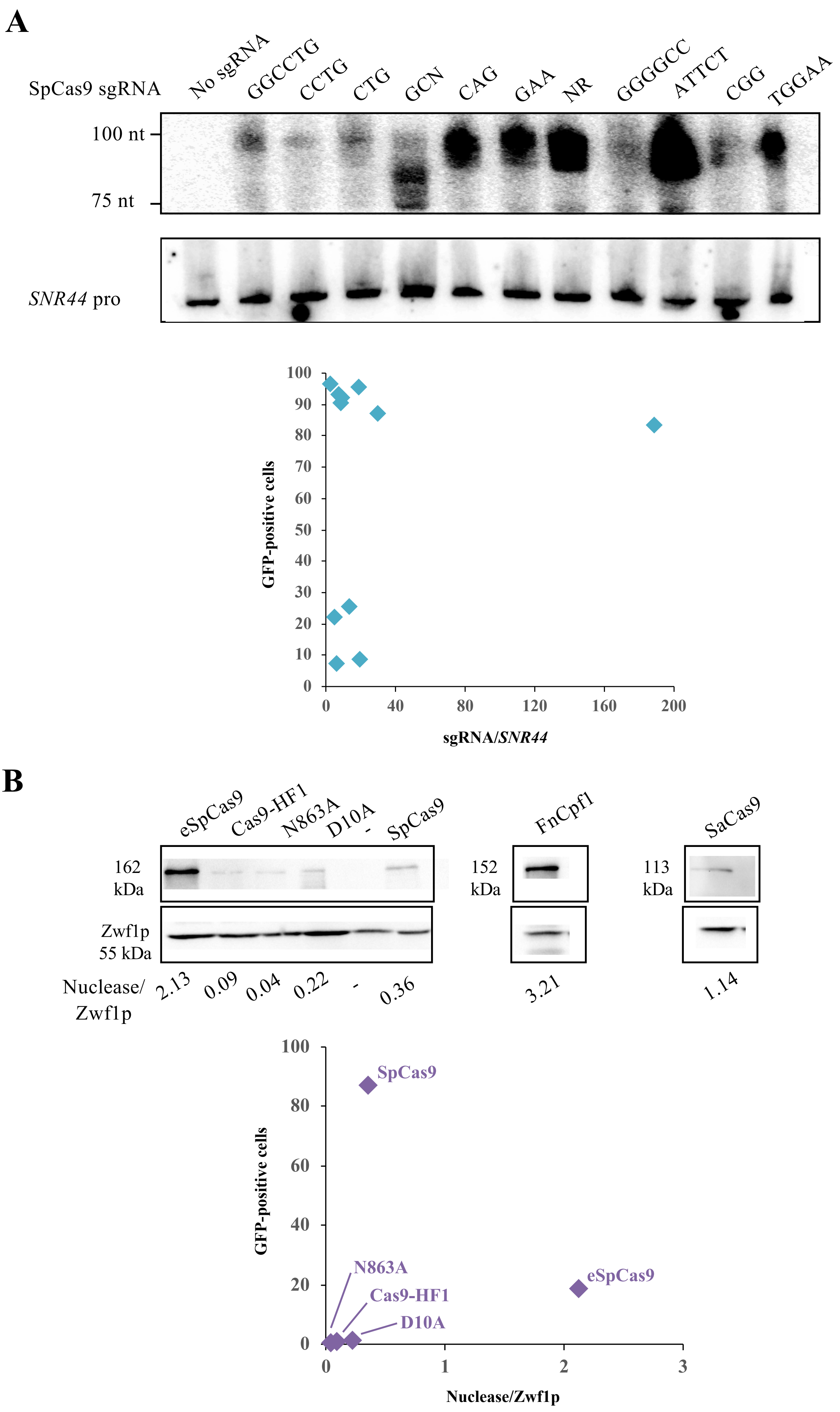
A
SpCas9 sgRNA
No sgRNA
GGCCTG
CCTG
CTG
GCN
CAG
GAA
NR
GGGGCC
ATTCT
CGG
TGGAA
100 nt
75 nt
SNR44 pro
GFP-positive cells
100
90
80
70
60
50
40
30
20
10
0
0
40
80
120
160
200
sgRNA/SNR44
B
eSpCas9
Cas9-HF1
N863A
D10A
-
SpCas9
FnCpf1
SaCas9
162 kDa
152 kDa
113 kDa
Zwf1p 55 kDa
Nuclease/Zwf1p
2.13
0.09
0.04
0.22
-
0.36
3.21
1.14
GFP-positive cells
100
80
60
40
20
0
0
1
2
3
Nuclease/Zwf1p
SpCas9
N863A
Cas9-HF1
D10A
eSpCas9
