# Supplementary material for: Differential efficacies of Cas nucleases on microsatellites involved in human disorders and associated off-target mutations
Source: Nucleic Acids Res. 2021 Jul 7;49(14):8120–34. doi: 10.1093/nar/gkab569 (PMC8373144; doi:10.1093/nar/gkab569)
Supplement: gkab569_Supplemental_Files [file gkab569_supplemental_files.zip › Supplementary Figures1-9.zip!/Supplemental Figure S6.pdf]

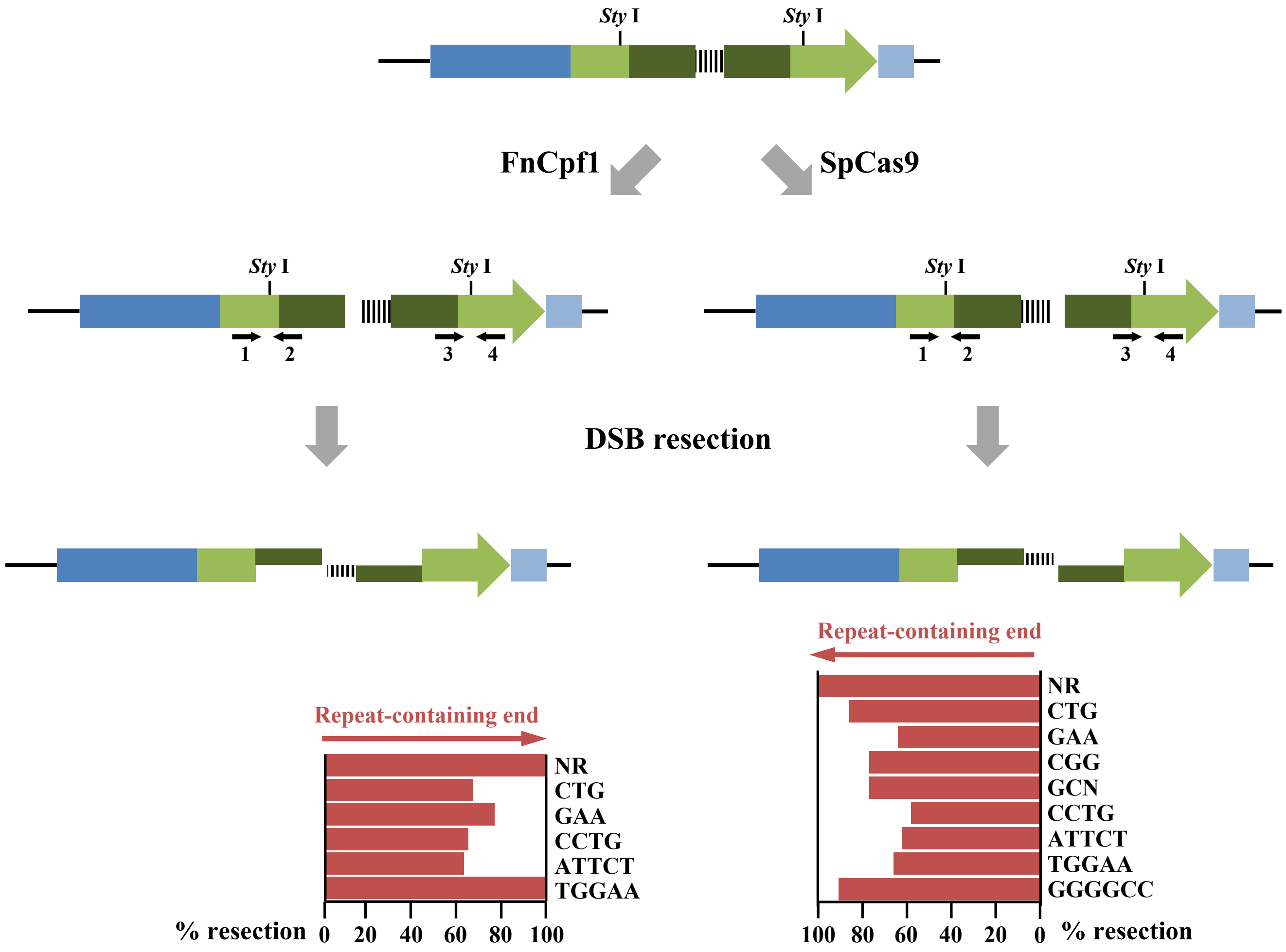

Sty I
Sty I
FnCpf1
SpCas9
Sty I
Sty I
1 2 3 4
Sty I
Sty I
1 2 3 4
DSB resection
Repeat-containing end
NR
CTG
GAA
CCTG
ATTCT
TGGAA
% resection
0 20 40 60 80 100
Repeat-containing end
NR
CTG
GAA
CGG
GCN
CCTG
ATTCT
TGGAA
GGGGCC
100 80 60 40 20 0
% resection
