# Supplementary material for: Differential efficacies of Cas nucleases on microsatellites involved in human disorders and associated off-target mutations
Source: Nucleic Acids Res. 2021 Jul 7;49(14):8120–34. doi: 10.1093/nar/gkab569 (PMC8373144; doi:10.1093/nar/gkab569)
Supplement: gkab569_Supplemental_Files [file gkab569_supplemental_files.zip › Supplementary Figures1-9.zip!/Supplemental Figure S8.pdf]

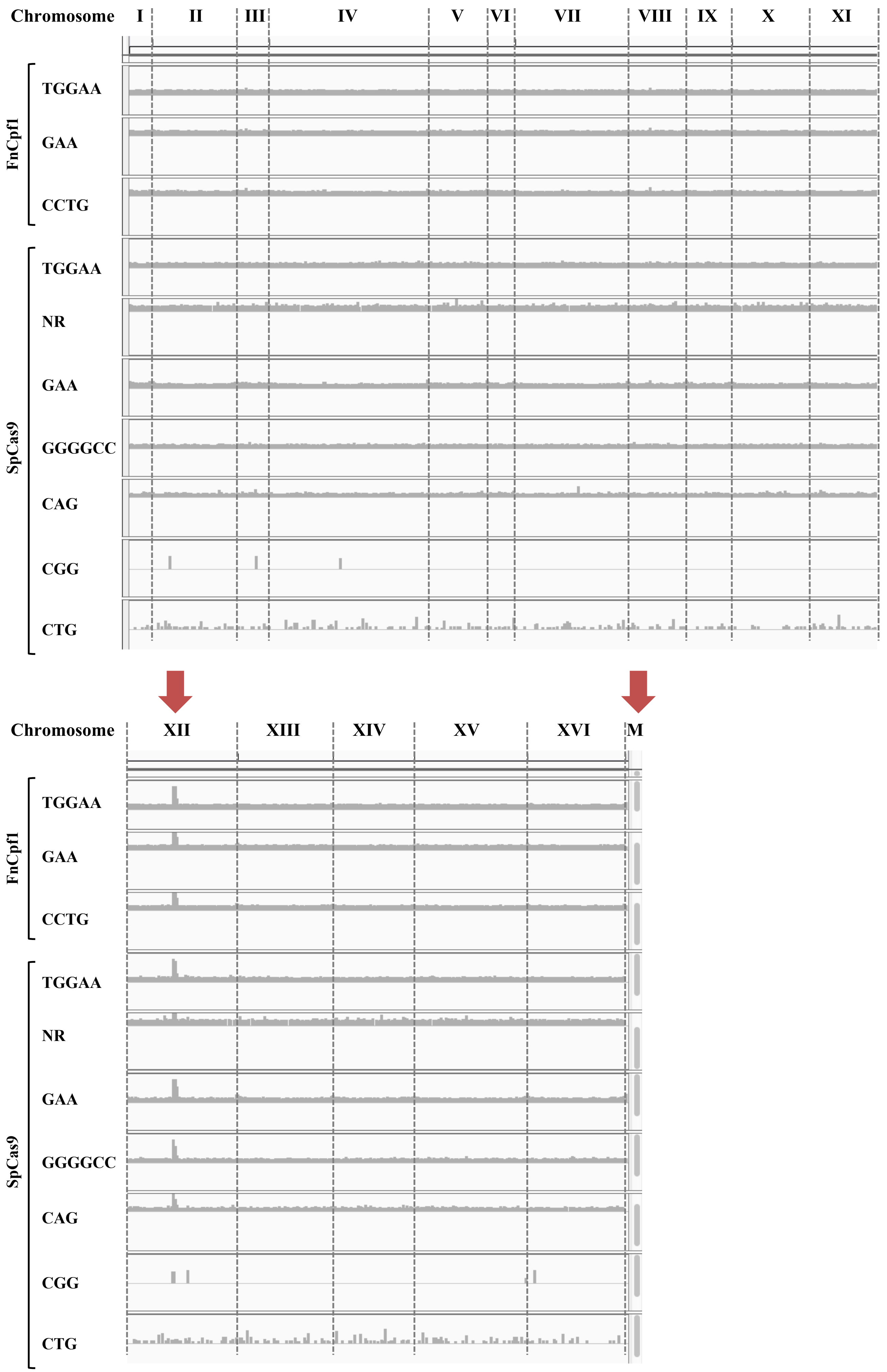
Chromosome
I
II
III
IV
V
VI
VII
VIII
IX
X
XI
FnCpf1
TGGAA
GAA
CCTG
SpCas9
TGGAA
NR
GAA
GGGGCC
CAG
CGG
CTG
Chromosome
XII
XIII
XIV
XV
XVI
M
FnCpf1
TGGAA
GAA
CCTG
SpCas9
TGGAA
NR
GAA
GGGGCC
CAG
CGG
CTG
