# Supplementary material for: Differential efficacies of Cas nucleases on microsatellites involved in human disorders and associated off-target mutations
Source: Nucleic Acids Res. 2021 Jul 7;49(14):8120–34. doi: 10.1093/nar/gkab569 (PMC8373144; doi:10.1093/nar/gkab569)
Supplement: gkab569_Supplemental_Files [file gkab569_supplemental_files.zip › Supplementary Figures1-9.zip!/Supplemental Figure S9.pdf]

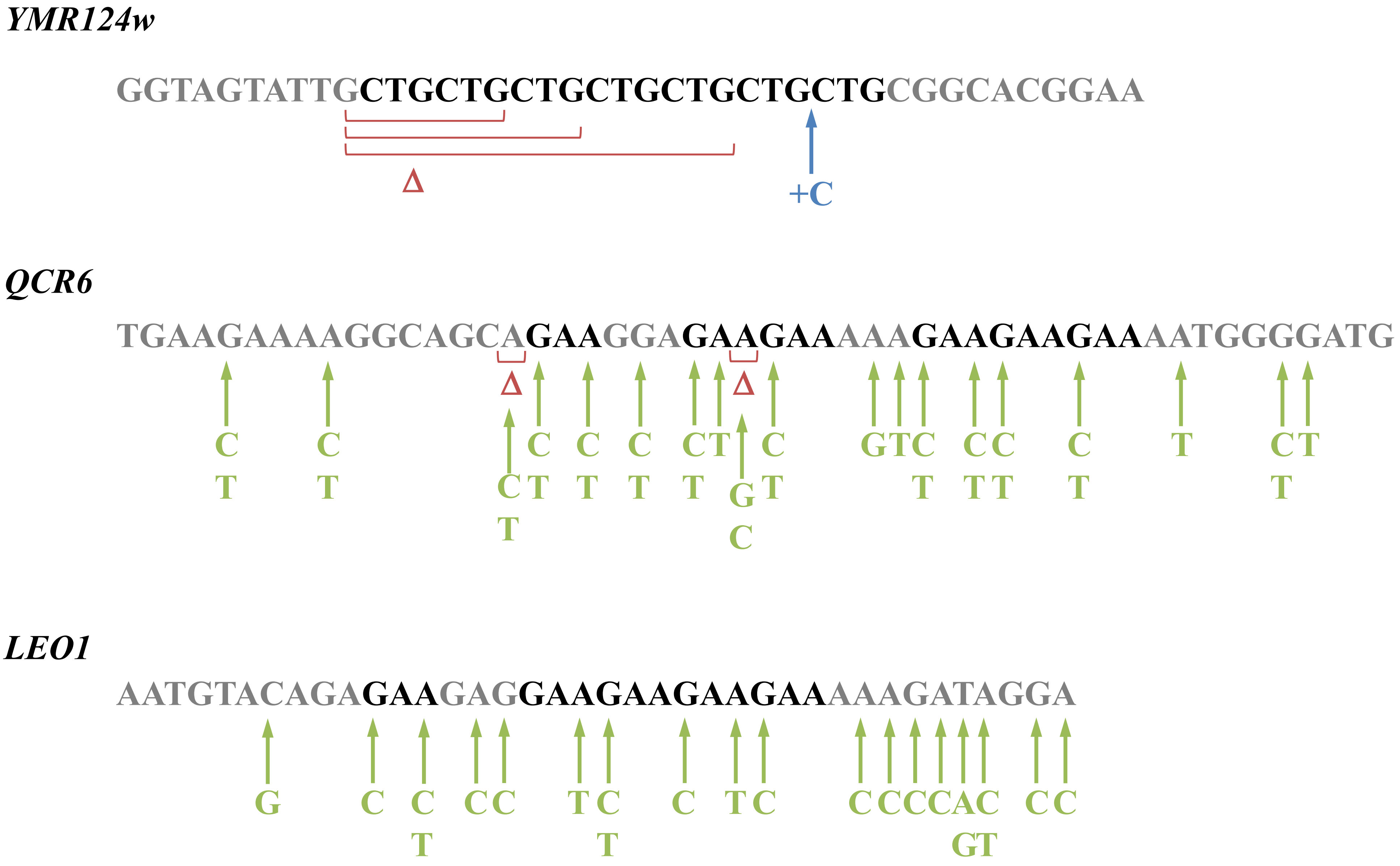

YMR124w
GGTAGTATTGCTGCTGCTGCTGCTGCTGCGGCACGGAA
Δ
+C
QCR6
TGAAGAAAAGGCAGCAGAAGGAGAAGAAAAAGAAGAAGAAAATGGGGATG
Δ
Δ
C C C C C CT C GTC CC C T CT
T T CT T T T GT T TT T T
T C
LEO1
AATGTACAGAGAAGAGGAAGAAGAAGAAAAAGATAGGA
G C C CC TC C TC CCCCAC CC
T T GT
