# Supplementary material for: Differential efficacies of Cas nucleases on microsatellites involved in human disorders and associated off-target mutations
Source: Nucleic Acids Res. 2021 Jul 7;49(14):8120–34. doi: 10.1093/nar/gkab569 (PMC8373144; doi:10.1093/nar/gkab569)
Supplement: gkab569_Supplemental_Files [file gkab569_supplemental_files.zip › Supplementary Tables1-6.zip!/Supplemental Table S4.docx]

**Supplemental Table S4: list of strains used in this study**

| **Name** | **Genotype** | **Origin** |
| --- | --- | --- |
| FYBL1-4D | *MAT*a *ura3*Δ851 *trp*Δ63 *leu2*Δ1 *his3*Δ200 *lys2*Δ202 | FYBL1 spore |
| LPY101 | *MAT*a *ura3*Δ851 *trp*Δ63 *leu2*Δ1 *his3*Δ200 *lys2*Δ202  *can1*Δ:: yEGFP(GGGGCC)_17_-*TRP1* | This study |
| LPY102 | *MAT*a *ura3*Δ851 *trp*Δ63 *leu2*Δ1 *his3*Δ200 *lys2*Δ202  *can1*Δ:: yEGFP(GAA)_33_-*TRP1* | This study |
| LPY103 | *MAT*a *ura3*Δ851 *trp*Δ63 *leu2*Δ1 *his3*Δ200 *lys2*Δ202  *can1*Δ:: yEGFP(GCN)_33_-*TRP1* | This study |
| LPY104 | *MAT*a *ura3*Δ851 *trp*Δ63 *leu2*Δ1 *his3*Δ200 *lys2*Δ202  *can1*Δ:: yEGFP(CGG)_33_-*TRP1* | This study |
| LPY105 | *MAT*a *ura3*Δ851 *trp*Δ63 *leu2*Δ1 *his3*Δ200 *lys2*Δ202  *can1*Δ:: yEGFP(GGCCTG)_17_-*TRP1* | This study |
| LPY106 | *MAT*a *ura3*Δ851 *trp*Δ63 *leu2*Δ1 *his3*Δ200 *lys2*Δ202  *can1*Δ:: yEGFP(TGGGAA)_20_-*TRP1* | This study |
| LPY107 | *MAT*a *ura3*Δ851 *trp*Δ63 *leu2*Δ1 *his3*Δ200 *lys2*Δ202  *can1*Δ:: yEGFP(ATTCT)_20_-*TRP1* | This study |
| LPY108 | *MAT*a *ura3*Δ851 *trp*Δ63 *leu2*Δ1 *his3*Δ200 *lys2*Δ202  *can1*Δ:: yEGFP(CCTG)_25_-*TRP1* | This study |
| LPY109 | *MAT*a *ura3*Δ851 *trp*Δ63 *leu2*Δ1 *his3*Δ200 *lys2*Δ202  *can1*Δ:: yEGFP(CAG)_33_-*TRP1* | This study |
| LPY110 | *MAT*a *ura3*Δ851 *trp*Δ63 *leu2*Δ1 *his3*Δ200 *lys2*Δ202  *can1*Δ:: yEGFP(CTG)_33_-*TRP1* | This study |
| LPY111 | *MAT*a *ura3*Δ851 *trp*Δ63 *leu2*Δ1 *his3*Δ200 *lys2*Δ202  *can1*Δ:: yEGFP(I-SceI cut site)-*TRP1* | This study |
| LPY112 | *MAT*a *ura3*Δ851 *trp*Δ63 *leu2*Δ1 *his3*Δ200 *lys2*Δ202  *can1*Δ:: yEGFP(I-SceI cut site)-*TRP1 dnl4*Δ::KANMX4 | This study |
| LPY113 | *MAT*a *ura3*Δ851 *trp*Δ63 *leu2*Δ1 *his3*Δ200 *lys2*Δ202  *can1*Δ:: yEGFP(I-SceI cut site)-*TRP1 pol32*Δ::KANMX4 | This study |
| LPY114 | *MAT*a *ura3*Δ851 *trp*Δ63 *leu2*Δ1 *his3*Δ200 *lys2*Δ202  *can1*Δ:: yEGFP(I-SceI cut site)-*TRP1 rad51*Δ::KANMX4 | This study |
| VMY104 | *MAT*α *ura3*Δ851 *leu2*Δ1 *his3*Δ200 *trp1*Δ63 *ade2*-opal *SUP4*::(CAG)80 *dnl4*Δ::KANMX4 | (Mosbach et al., 2018) |
| VMY432 | *MAT*α *ura3*Δ851 *leu2*Δ1 *his3*Δ200 *trp1*Δ63 *ade2*-opal *SUP4*::(CAG)80 *pol32*Δ::KANMX4 | (Mosbach et al., 2018) |
| VMY551 | *MAT*a *ura3*Δ851 *leu2*Δ1 *his3*Δ200 *trp1*Δ63 *ade2*-opal *SUP4*::(CAG)70 *rad51*Δ::KANMX4 | (Mosbach et al., 2018) |
